# Supplementary material for: Simulated Evolution of Protein-Protein Interaction Networks with Realistic Topology
Source: PLoS One. 2012 Jun 29;7(6):e39052. doi: 10.1371/journal.pone.0039052 (PMC3387198; doi:10.1371/journal.pone.0039052)
Supplement: File S1 — Supporting information text. (PDF) [file pone.0039052.s001.pdf]

Supporting Information

Subfunctionalization

One model for the fate of duplicate genes is *subfunctionalization* (SF). In SF, the original and the duplicate genes are both free to lose their redundant functions, so they can evolve freely until they exactly reproduce the ancestral function [92]. The post-duplication divergence in our model is similar in spirit to SF, but it differs in two significant ways: (1) in our model, the link loss is completely asymmetric, and (2) a fraction $(1-\phi)$ of the redundant links are retained, so, unlike SF, not all of the redundancy is eliminated. For the first point, empirical evidence suggests that the divergence is asymmetric [31], although the assumption of *complete* asymmetry would likely need to be revisited to build a finer-grained model. Second, genetic regulatory networks have been shown to be robust to random link deletions, indicating that these networks retain some degree of redundancy [93–95]. *In silico* evidence suggests that a more accurate picture may be of a transient period of functional divergence, followed by prolonged neofunctionalization, resulting in only a partial loss of redundancy [96]. This is consistent with our model.

Large-scale duplications

Our model does not explicitly consider simultaneous duplication of multiple genes (chromosomal duplications, whole genome duplications, etc.). However, as shown in Table 1, duplication rates in our model are considerably higher than neofunctionalization rates, so that, on average, there are multiple duplications per neofunctionalization event. Sequential duplications of this type may be thought of as an (imperfect) representation of multi-gene duplications. The advantage of this approach is that we do not require separate rates for each duplication scale (one could imagine an extremely detailed model which included separate rates for gene duplication, gene-pair duplication, gene-triplet duplication, etc.). The downside is that our implementation of larger-scale duplications will generally include some genes which have been duplicated multiple times, and others which have not been duplicated at all. A potential mitigating factor is that the rate of gene loss (and evolution in general) following genome duplication is very high [30, 55], so even a completely faithful large-scale duplication would likely be altered within short order.

Randomness conjecture

We describe here a test of our randomness conjecture, $q = 1/N$. The implication is that the average number of NE links per protein should be independent of N. Another possibility is that q is constant, implying that the number of NE links per protein is proportional to N. To test this, we compared NE rates in the human and yeast PPI networks. The total number of proteins in humans is estimated to be 22740 [97] and in yeast, 5616 [98]. The number of mutations to coding DNA is approximately 0.004/genome/replication in humans and 0.0027/genome/replication in yeast [99]. If the number of new links is proportional to N, then, based on the number of proteins and the mutation rate, there should be roughly 600% more links created by NE in humans than in yeast. However, by counting the number of nonredundant interactions in duplicate gene pairs, it has been shown empirically that the average number of links created by NE per protein is only about 8% higher in humans than in yeast [51]. These results support the conjecture that the probability for a protein to receive a new link via point mutation is approximately independent of N, as previously noted in [46]. Due to the finite copy number of proteins, as well as the compartmentalization of eukaryotic cells, we regard it as unlikely that proteins will simultaneously acquire multiple links to targets in different locations in the cell, or which are involved in divergent biological processes.

Assimilation is driven by single-interface proteins

Closely related to the randomness conjecture is the number of 'extra' links created by each assimilation event, which should also independent of N. Proteins with multiple interaction partners may interact with their partners simultaneously (so-called 'party hubs') or at different times/locations ('date hubs') [100]. Since they bind to several partners simultaneously, party hubs typically have multiple binding sites, each specific to one binding partner [101]. This specificity suggests that a protein which evolved the capability to bind to a party hub would be unlikely to undergo assimilation. By contrast, the binding sites of date hubs are often disordered regions which are able to form transient interactions with multiple partners [102, 103]. If a protein evolves the capability to bind to a date hub, it is likely to share the physical characteristics of the hub's neighbors, leading to assimilation. However, to avoid competition for the same binding site, the interaction partners of date hubs tend not to be coexpressed [101]. One consequence of this is that assimilating proteins will likely only bind one of the target protein's neighbors – whichever neighbor happens to be present at that time and place. Although the capability to bind to the hub protein's other neighbors may initially be present, these will presumably remain unused in the cell. Our expectation is that the assimilating protein will therefore be unlikely to retain this capability, as it evolves. Similarly, only a single extra link should be generated at the second-neighbor level, third-neighbor level, etc. Consistent with the evidence discussed above, the number of links created by assimilation is approximately independent of the total network size. Party hubs typically are centrally-located within modules, while date hubs often function to stitch together large-scale modules in the cell. It may be that duplication-only models are unrealistically fragmented (Table 2) because their modules are not properly attached with date hubs; instead, the modules are disconnected components.

Domain shuffling and assimilation

One example of a known biological mechanism which should lead to assimilation is domain shuffling, the copy-and-pasting of part of one protein into another [104, 105]. The neofunctionalization mechanism described here is quite general, and includes domain shuffling, among other methods of PPI creation. A PPI formed via domain shuffling will often be the result of a binding site duplication. Assuming the binding is due to simple surface similarity, the initial link will be to the protein which had its domain copied. The likelihood of binding to neighbors of the original protein should depend only on the probability that each interaction is due to surface similarity because the copied binding site will be identical to the original.

The role of domain shuffling in assimilation raises the question of whether domains should be modeled explicitly, rather than representing proteins as integral units. Previous work indicates that overall PPI network topology is robust to the details of domain shuffling [106]. Moreover, while proteins which have experienced domain shuffling have a higher average degree than other proteins, high- and low-degree proteins are equally likely to acquire new interactions this way [107]. Because the creation of new links by domain shuffling should be topologically very similar to the creation of new links by other neofunctionalization events, we believe our model is a reasonable implementation of this mechanism, as it applies to the evolution of network topology.

Network rewiring

Some higher-order features of the network are simply a result of its degree sequence, and other features might be important in their own right. As discussed in [61], it is possible to isolate the effects of the degree sequence by 'rewiring' (detaching then reattaching links) the network at random, subject to the restriction that the degree sequence must be preserved. If a property contains extra information about the network's structure, then it should be different in the rewired network. On the other hand, if the network is rewired many times, and the property is always the same, then it is likely to just be a result of the

degree sequence. We used a script downloaded from `http://www.cmth.bnl.gov/~maslov/matlab.htm` to randomly rewire the empirical network $4K_{\text{data}}$ times. As expected, modularity is decreased by random rewiring. Upon rewiring, we find $Q = 0.603 \pm 0.002$ in humans, $Q = 0.590 \pm 0.003$ in yeast, and $Q = 0.722 \pm 0.007$ in flies (median $\pm$ standard deviation from 50 repeats of the rewiring algorithm). Rewiring also shrinks the diameters of PPI networks to $D = 13 \pm 0.9$ in humans, $D = 12 \pm 1.0$ in yeast, and $D = 15 \pm 1.1$ in flies. These results suggest that these features contain important structural information about the network, and are not merely consequences of the degree sequence.

One reason we are interested in calculating Q and D is simply to check that the values are comparable between the simulated and experimental networks. However, on a more qualitative level, we would also like to have some idea of what the threshold is for a network to be considered 'modular' or 'small-diameter'. The rewired Q and D values are useful because these features are dependent on the size of the network (number of nodes N and links K); given an identical network construction method, Q and D will generally be different in sparse versus dense networks. We use these Q and D values as baseline values with which the experimental and simulated networks can be compared; we considered Q and D values differing from the rewired values by more than a standard deviation to be significantly different.

Eigenvalues

The connectivity of a network can be expressed by its *adjacency matrix*, an $N \times N$ matrix $\mathbf{A}$, in which the entries A_{ij} equal 1 if a link exists between proteins i and j, and 0 otherwise. If $\mathbf{A}$ is normalized by column, then the entries describe the rates of a transition from i to j in one time step. The distribution of eigenvalues $p(\lambda)$ is called the network's *spectrum* (Figure S7). This matrix and its eigenvalues can be interpreted in terms of a process in which a random walker starts on one node i, and, over a series of time steps, reaches another node j. Intuitively, this can be thought of as a signal propagation rate: if one protein is affected by an external signal, how long does it take that signal to diffuse through the network? The eigenvalues λ of this 'walk matrix' describe the rate at which a random walk on the network reaches steady-state. The second-largest eigenvalue (λ_2) determines the rate of convergence of the random walk (Figure S3). A larger value of λ_2 indicates a slower signal propagation rate.

Error tolerance

We measured the 'error tolerance' as described in [29]: we examined the decrease of f_1 when nodes (and their accompanying edges) were deleted from the network either (1) at random, or (2) according to their degree, starting with the most well-connected node. Results for the simulated and experimental networks were very similar (Figure S8).

Simulation length: early versus late evolution

The total time elapsed during our simulations varies considerably, with yeast and human simulations running about 1 to 2 billion years, and the fly simulations about 5 billion years. This is compared to the rough estimate of 3.5 billion years since the origin of life on Earth [108]. The exceptionally long duration of the fly simulations are due to the very low gene duplication rate ($d = 0.001$/gene/Myr). The aim of our model is to describe the evolution of PPI networks with all their present-day machinery. Gene duplication, in the form in which it exists today, certainly would not have existed at the origin of life! The initial state in our model consists of two interacting proteins. Biologically, these are two polypeptides (or, more likely, RNA molecules) in a pre-biotic soup, that happen to interact in a way that is mutually beneficial. Each of these molecules has the ability to replicate. This autonomous replication of individual proteins corresponds to 'gene duplication' in the very early stages of evolution. However, this is a very different conceptual underpinning for the duplication mechanism, and it seems unlikely to share the present-day values of the duplication rate. Because, in the early stages of evolution, each time

step represents a very long duration in real time, it is likely that this accounts for the discrepancy in total time elapsed.

Empirical data

We downloaded large-scale data sets from BioGRID [109], and used the Wilcoxon rank-sum test to compare aggregate statistical features across various experimental types in yeast (*S. cerevisiae*) and humans (*H. sapiens*) [100]. As expected, we found that data obtained by affinity capture was significantly different than pairwise experimental data (primarily yeast two-hybrid and *in vitro* complexation), as the affinity capture interactions represent entire complexes, which is somewhat different information than the pairwise interactions we are attempting to capture using our model. However, more surprisingly, the only feature to show significant agreement between pair-wise techniques was the eigenvalue distribution of the walk matrix ($P > 0.05$). Further sub-dividing the individual techniques into smaller data sets containing only results obtained in single experiments, we discovered that, again, only the spectra agreed between different screens.

Note that, due to the small size of the fly network, there may be too many missing links to obtain an accurate description the network's large-scale topology. Although, by appropriate parameter tuning, our model is able to accurately reproduce the fly network, it is possible that different parameters will be required to match the fly network once it becomes more fully characterized experimentally. The data sets considered here do not include interactions which are enabled through post-translational modifications. Although these data sets are far from complete, and may be susceptible to false-positive detections, these appear to be the most accurate data available at the present time.

Fitting functions

The degree distribution obeys a power law in its tail, $p(k) \sim k^{-\gamma}$ [91], with $\gamma \approx 3$ (Table S1), implying that hub proteins are more common than would be expected for a randomly connected network, which would have an exponentially decaying $p(k)$. The closeness distribution $p(\ell)$ is approximately Gaussian, with mean 0.17 and standard deviation 0.03 in humans, mean 0.19 and standard deviation 0.03 in yeast, and mean 0.13 and standard deviation 0.03 in flies. Closeness is a measure of distance, indicating that the distances within the network are essentially a random walk in 'node space'. The betweenness distribution also follows a power law in its tail. This is an indication of modular structure, due to the overrepresentation of 'bridge' proteins, relative to a randomly connected network.

All species examined show a power law decay in clustering coefficient as a function of degree, $C \sim k^{-\xi}$. Poorly-connected proteins therefore tend to have *higher* clustering coefficients, meaning that a greater fraction of their neighbors are mutually connected.

Disassortative mixing was quantified for the yeast PPI network in [61] as a power law *decrease* in median neighbor degree, $n \sim k^{-\delta}$. This is consistent with our data, although the very small estimated value of $\delta = 0.32$ indicates only a slight negative relation (Table S1). Interestingly, $\delta = 0$ in both human and fly networks, indicating that disassortativity may be a trait unique to the yeast network.

Principal component analysis

We examined six features which calculate a value for each node in the network: degree centrality, clustering coefficients, closeness centrality, eigenvalue spectrum, betweenness centrality, and mean nearest-neighbor degree. To quantify the independence of these features, we used principal component analysis (PCA) [110]. Each feature assigns a value to each node in the network, giving a $6 \times N$ data matrix, where each row represents a feature (signal), and each column is a node (sample). We subtract the mean and divide by the standard deviation of each row. This results in a standardized data matrix, denoted by $\mathbf{Y}$.

The 6×6 correlation matrix for each species is defined as $\mathbf{C} \equiv \frac{1}{N-1}\mathbf{Y}\mathbf{Y}^{\mathrm{T}}$:

$$\mathbf{C}_{\mathrm{h}} = \begin{bmatrix} 1 & 0.10 & 0.87 & 0.56 & 0.02 & -0.12 \\ 0.10 & 1 & -0.04 & 0.09 & 0.01 & 0.03 \\ 0.87 & -0.04 & 1 & 0.44 & -0.01 & -0.08 \\ 0.56 & 0.09 & 0.44 & 1 & 0.00 & 0.34 \\ 0.02 & 0.01 & -0.01 & 0.00 & 1 & -0.02 \\ -0.12 & 0.03 & -0.08 & 0.34 & -0.02 & 1 \end{bmatrix}, \tag{4}$$

$$\mathbf{C}_{\mathrm{y}} = \begin{bmatrix} 1 & 0.03 & 0.91 & 0.43 & -0.02 & -0.21 \\ 0.03 & 1 & -0.06 & 0.04 & 0.01 & 0.03 \\ 0.91 & -0.06 & 1 & 0.39 & -0.01 & -0.14 \\ 0.43 & 0.04 & 0.39 & 1 & -0.03 & 0.34 \\ -0.02 & 0.01 & -0.01 & -0.03 & 1 & -0.04 \\ -0.21 & 0.03 & -0.14 & 0.34 & -0.04 & 1 \end{bmatrix}, \tag{5}$$

$$\mathbf{C}_{\mathrm{f}} = \begin{bmatrix} 1 & 0.15 & 0.62 & 0.36 & -0.11 & -0.15 \\ 0.15 & 1 & -0.08 & 0.06 & -0.10 & -0.02 \\ 0.62 & -0.08 & 1 & 0.40 & 0.02 & -0.16 \\ 0.36 & 0.06 & 0.40 & 1 & 0.00 & 0.30 \\ -0.11 & -0.10 & 0.02 & 0.00 & 1 & -0.05 \\ -0.15 & -0.02 & -0.16 & 0.30 & -0.05 & 1 \end{bmatrix}. \tag{6}$$

The entries of each $\mathbf{C}$ are (from left-to-right, and top-to-bottom): degree centrality, clustering coefficients, betweenness centrality, closeness centrality, eigenvalue spectrum, and mean nearest-neighbor degree. Many of the off-diagonal elements of the $\mathbf{C}$ matrices are close to zero, suggesting that the features are to a large extent independent of one another.

To perform PCA, we diagonalized each correlation matrix,

$$\mathbf{C} = \mathbf{S}\boldsymbol{\Lambda}\mathbf{S}^{\mathrm{T}}, \tag{7}$$

where $\boldsymbol{\Lambda}$ is a diagonal matrix of eigenvalues and $\mathbf{S}$ has the eigenvectors of $\mathbf{C}$ as its columns. As shown in Figure S9, the degree and betweenness show similar loadings on the first two principal components, reflecting the nearly linear relation between these centrality scores (Figure S10).

The eigenvalue matrices $\boldsymbol{\Lambda}$ are given by:

$$\boldsymbol{\Lambda}_{\mathrm{h}} = \begin{bmatrix} 2.27 & & & & & \\ & 1.23 & & & & \\ & & 1.02 & & & \\ & & & 0.98 & & \\ & & & & 0.40 & \\ & & & & & 0.11 \end{bmatrix}, \tag{8}$$

$$\boldsymbol{\Lambda}_{\mathrm{y}} = \begin{bmatrix} 2.20 & & & & & \\ & 1.30 & & & & \\ & & 1.01 & & & \\ & & & 0.98 & & \\ & & & & 0.43 & \\ & & & & & 0.08 \end{bmatrix}, \tag{9}$$

$$\mathbf{\Lambda}_{\mathrm{f}} = \begin{bmatrix} 1.95 & & & & & \\ & 1.24 & & & & \\ & & 1.13 & & & \\ & & & 0.91 & & \\ & & & & 0.44 & \\ & & & & & 0.32 \end{bmatrix}. \tag{10}$$

(Zeros have been suppressed for clarity.) The fraction of variance explained by the ith principal component is given by $\Lambda_{ii}/\sum_j \Lambda_{jj}$. The closer the number of components required to explain most of the variance is to the total number of input signals, the more independent the signals are. In yeast and humans, 4 components are required to explain 90% of the variance; in fruit flies, it requires 5 components. Linear transformations are able to only modestly reduce the dimensionality of the problem, suggesting that each feature contributes unique information about the network's structure. This does not, of course, rule out the possibility of the existence of other independent, informative features, a far more complicated question which is outside the scope of this current work.

Sensitivity analysis

The DUNE model has four parameters. One parameter, the DU rate d, is estimated from empirical data. The other three are adjustable parameters: the NE rate μ, the divergence probability ϕ, and the assimilation probability a. To gain a better understanding of how these parameters affect the final network structure, starting with each organism's set of parameters, we systematically adjusted all 4 parameters. Results are shown in Figure S11.

As expected, the divergence parameter ϕ was positively correlated with both the modularity Q and the diameter. When $\phi \approx 0$, the network quickly reaches a fully-connected state, where all proteins are linked to all other proteins. Consequently, the network is not organized into modules, and all distances in the network are equal to 1. The 'thinning out' of duplicate links is therefore essential to generate non-trivial network features. The opposite occurs when the NE rate μ is too low: $f_1 \approx 0$, and the network evolves towards a completely disconnected state.

Why does gene duplication lead to modularity? Consider an initially uniform network. When a node is duplicated at random, this causes the original node, the copy, and their immediate neighbors to share more links internally than they do with the rest of the network. Subsequent duplications amplify this effect: if a node that has 10 links within a module but only 2 external links is duplicated, there are now 20 internal and 4 external links (prior to post-duplication divergence).

Interestingly, Q has a weak negative correlation with the assimilation parameter a. When a is large, the probability to link to distant neighbors of the target protein is relatively high, and mutated proteins have a non-negligible chance to generate links to proteins outside of their target's pathway. This causes modules to blur at the edges; their member proteins will share a higher number of links to other modules than for a low a network. Although the modularity is reduced for a high a network, it does not disappear entirely. Similarly, there is a sharp decrease in Q as the NE rate surpasses the DU rate, indicating the important role of the DU mechanism in modular organization.

Diameter is also negatively correlated with a. When a single NE event has a significant chance to generate links to the target protein's neighbors, this tends to reduce the overall separation of proteins in the network.

Comparison to other models

Our model is rooted in previous modeling efforts. The basic framework for our model combines the gene duplication mechanism described in [29] with a link creation mechanism inspired by [85]. The principal difference between our model and previous models is that our model considers duplication and mutation simultaneously. The previous models we examined attempted to construct the PPI network from a single

mechanism. Another significant difference is our assimilation mechanism. To the best of our knowledge, previous work has not explicitly modeled proteins integrating into biological pathways.

We compare the DUNE model to four models previously proposed for PPI networks. Two were evolutionary models: (1) the Vázquez model of DU followed by rapid loss-of-function mutations [29] and (2) the Berg 'link dynamics' model of point mutations coupled with a PA-like 'rich-get-richer' rule for assigning new interactions [85]. (A slightly different DU model is presented by Pastor-Satorras [111]. However, because the Vázquez model has been shown to be a better fit to experimental data [112], we have limited our DU-only comparison here to the Vázquez model.) Two others were static models (models of present-day networks that do not simulate the network's evolutionary path) that consider the primary organizing principle to be nonspecific interactions between proteins: (1) random geometric (RG), a mathematical model where proteins are randomly scattered in a 2 to 4 dimensional box, and any proteins close enough to one another form an interaction [89] and (2) the 'MpK' desolvation model, which assigns interactions based on proteins' exposed hydrophobic surface areas [52]. For reference, we also calculated results for an Erdős-Rényi (ER) random graph with N and $\langle k \rangle$ set by the data [90].

These models were originally validated against different features of the empirical network, making it difficult to directly compare them. To characterize these models in greater detail, we coded each of these models, and ran 50 simulations of each model with identical parameters and starting conditions. Using Matlab, we coded the Vázquez [29], Berg [85], RG [89], and MpK [52] models as described in the original papers. Since each model was originally parametrized for older yeast PPI data sets, we re-optimized the parameters for our yeast data as follows. We used a Monte Carlo simulation to adjust each model's parameters to minimize the total symmetric mean absolute percentage error values (SMAPE; see below) for the yeast HitPredict data set.

For the Vázquez model, we used a value of 0.582 for the post-duplication divergence probability, and a value of 0.083 for the dimerization probability. As noted by previous authors, duplication-only simulations produce networks which are extremely fragmented [82]. We observed that the Vazquez simulations typically had around 20% of their nodes in the largest connected component (Table 2). Since most of the network features we examined are limited to the largest component, in order to make a reasonable comparison of the Vazquez simulation results to the data, we allowed the simulated network to grow until its number of links met or exceeded 5 times the number of links in the data, $K \geq 5K_{\text{data}}$. Since the largest component is not always exactly 20% of the total nodes, this stopping condition is somewhat arbitrary; however, results for this model seem robust to small changes in the stopping condition. For the Berg model, we used the empirically estimated duplication rate of 0.01/gene/Myr, and found best-fit values of 24.5/gene/Myr for the mutation rate, and $N_{\text{data}} - 98$ proteins for the initial network size. For the RG model, we used a $45.5 \times 45.5 \times 45.5$ 'box' with a maximum interaction radius of 3.92. For the MpK model, the number of exposed surface residues was 19, the fraction of exposed hydrophobic residues was $M = 0.230 \pm 0.110$ (mean $\pm$ standard deviation), and the best-fit linear equation relating M to the binding threshold was $1.09M + 1.04$.

The Vázquez simulations were initialized with 2 connected nodes, and the simulation was allowed to run until $K \geq 5K_{\text{data}}$. The Berg simulations were initialized with $N_{\text{data}} - 98$ randomly connected nodes, then run until $N = N_{\text{data}}$. The RG and MpK models (which are not evolutionary models and therefore create the network all at once) were set up as described in the original papers.

To characterize the networks, we computed several network properties:

Single-value: modularity Q, diameter D, fraction of nodes in the largest component f_1, global clustering coefficient $\langle C \rangle$, and average protein degree in the largest component $\langle k \rangle$ (Table 2)

Distributional: degree $p(k)$, betweenness $p(b)$, closeness $p(\ell)$, eigenvalue $p(\lambda)$, and nearest-neighbor degree $p(n)$ distributions

Scatter plot: closeness vs. degree $\ell(k)$, clustering coefficient vs. degree $C(k)$, betweenness vs. degree $b(k)$, median nearest-neighbor degree vs. degree $n(k)$, and error tolerance curves

and compared these features to those of empirical data from yeast. As shown in Figure S12, we found that none of the previous models capture the full set of network properties.

To quantify agreement with the data for non-single-value features, we calculated the symmetric mean absolute percentage error (SMAPE) between simulation and experiment [113, 114]:

$$\text{SMAPE} = \frac{1}{Y}\sum_{i}^{Y}\frac{\left|y_i - y_i^{\text{data}}\right|}{y_i + y_i^{\text{data}}}, \tag{11}$$

where Y is the number of data points, and y_i and y_i^{data} denote the ith point of the response variable (Table S2) in the simulated and experimental data, respectively. For the distributional features, Y is the number of bins (arbitrarily chosen to be 100) minus the number of bins in which $y_i + y_i^{\text{data}} = 0$. For non-distributional (scatter plot) features, Y is the number of k values with values for both simulation and experiment. There are many possible measures of accuracy (such as the widely-used root mean squared error); we used SMAPE for two reasons. First, because it relies on absolute value, SMAPE does not over-emphasize the impact of outliers. Second, dividing by $y_i + y_i^{\text{data}}$ ensures that the magnitude of the response variable does not overwhelm the sum. This is significant for the non-distrbutional features. For example, in a plot of betweenness vs. degree (Figure S10), we are just as interested in the overlap of the low-betweenness, low-degree region of the curve as we are with the high-betweenness, high-degree region. SMAPE values are collected in Table S2. As shown in Tables 2 and S2, while previous models accurately reproduce certain features of the PPI network, only the DUNE model provides a reasonable across-the-board fit.
