# Supplementary figures and images for: Simulated Evolution of Protein-Protein Interaction Networks with Realistic Topology
Source: PLoS One. 2012 Jun 29;7(6):e39052. doi: 10.1371/journal.pone.0039052 (PMC3387198; doi:10.1371/journal.pone.0039052)

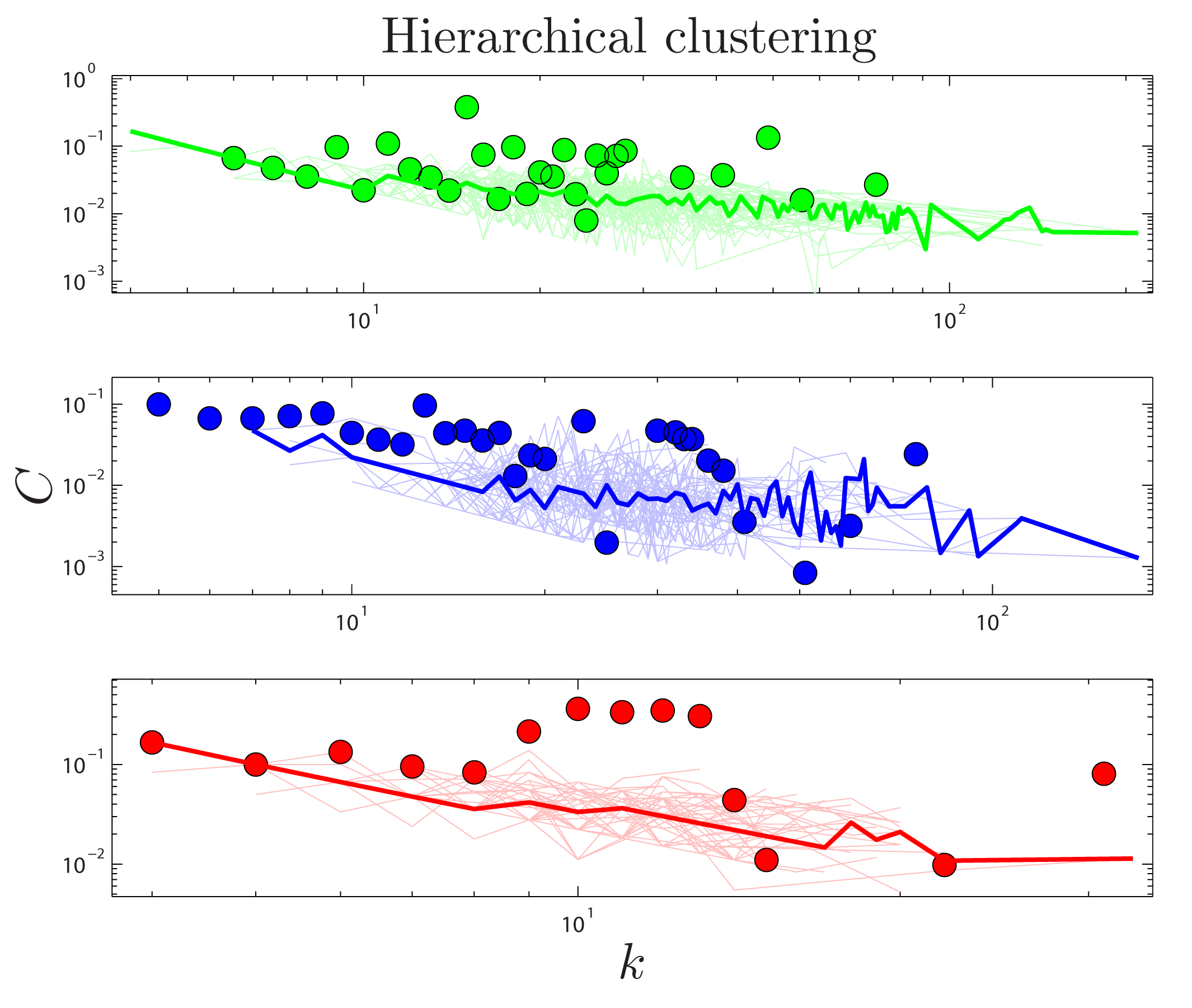

Supplement: Figure S1 — Hierarchical clustering. Median clustering coefficient vs. degree in human (green), yeast (blue), and fly (red). Heavy lines are the median values from 50 simulations, and light lines are results of individual simulations. (TIF) [file pone.0039052.s002.tif]

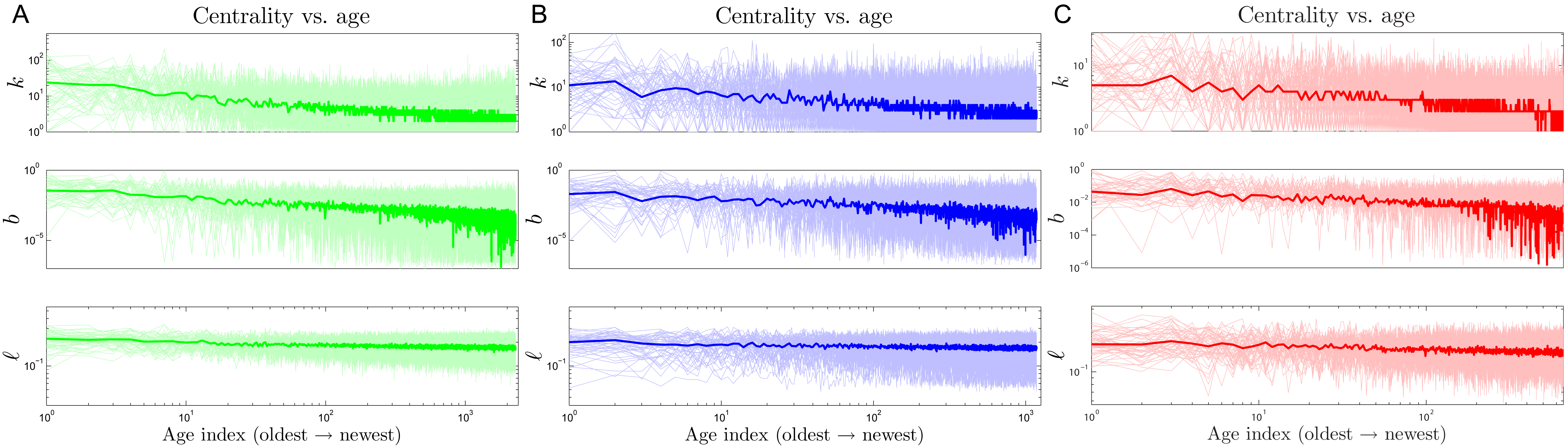

Supplement: Figure S2 — Older proteins are more central. Simulations of a protein’s age index (time since introduction into the network) vs. degree (), betweenness (), and closeness () centrality, for human (green), yeast (blue), and fly (red). The oldest proteins are on the left in this figure, and the proteins get younger moving to the right. There is an approximately monotonic increase in centrality with age. (TIF) [file pone.0039052.s003.tif]

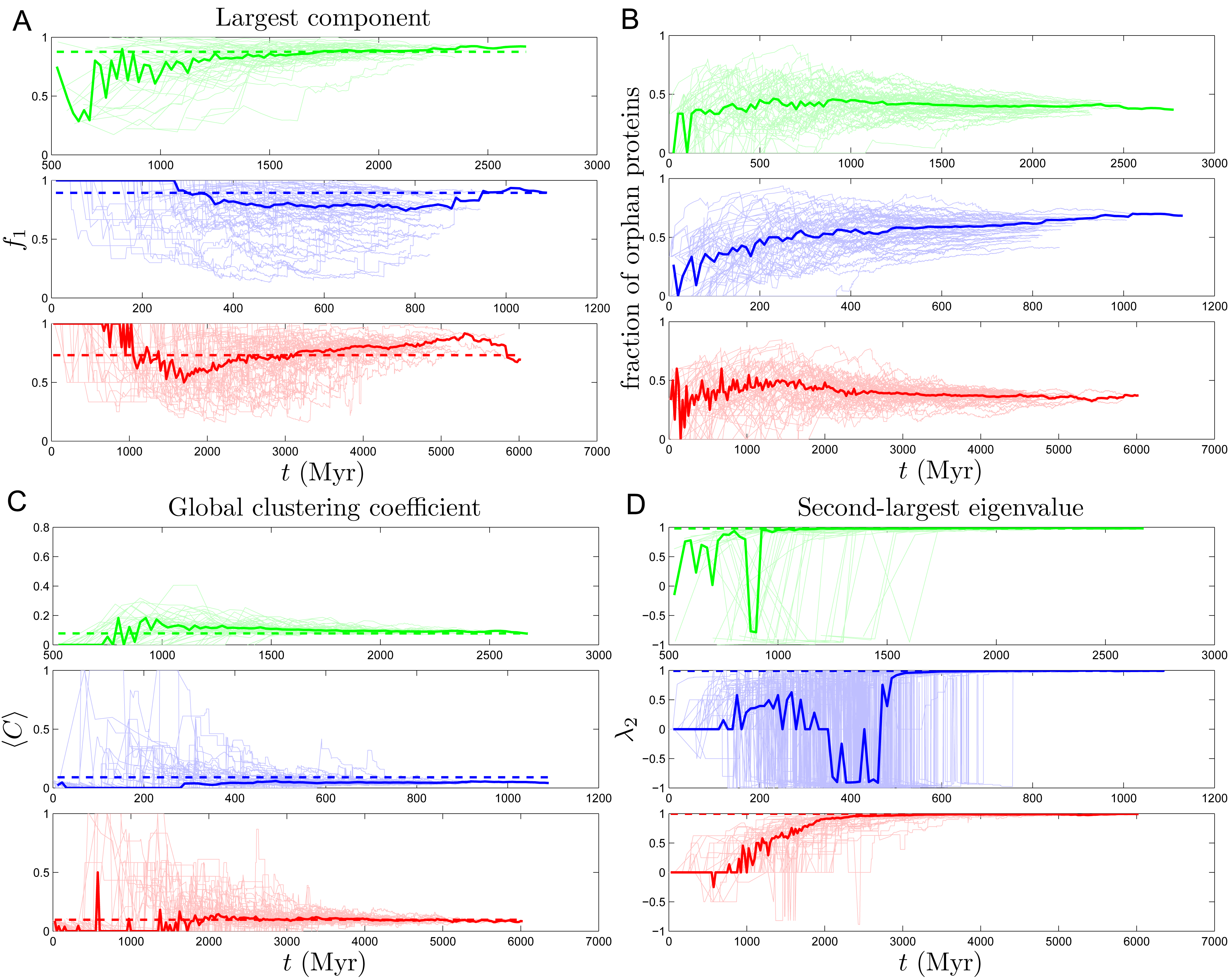

Supplement: Figure S3 — Dynamical features. Shown are the evolution of (A) the largest component size, (B) the fraction of orphan proteins, (C) the global clustering coefficient, and (D) the second-largest eigenvalue of the walk matrix, in human (green), yeast (blue), and fly (red). Light lines indicate the evolutionary trajectories of 50 individual simulations, and the heavy line is the median value. Empirical data values are shown as a dashed line, where available. (TIF) [file pone.0039052.s004.tif]

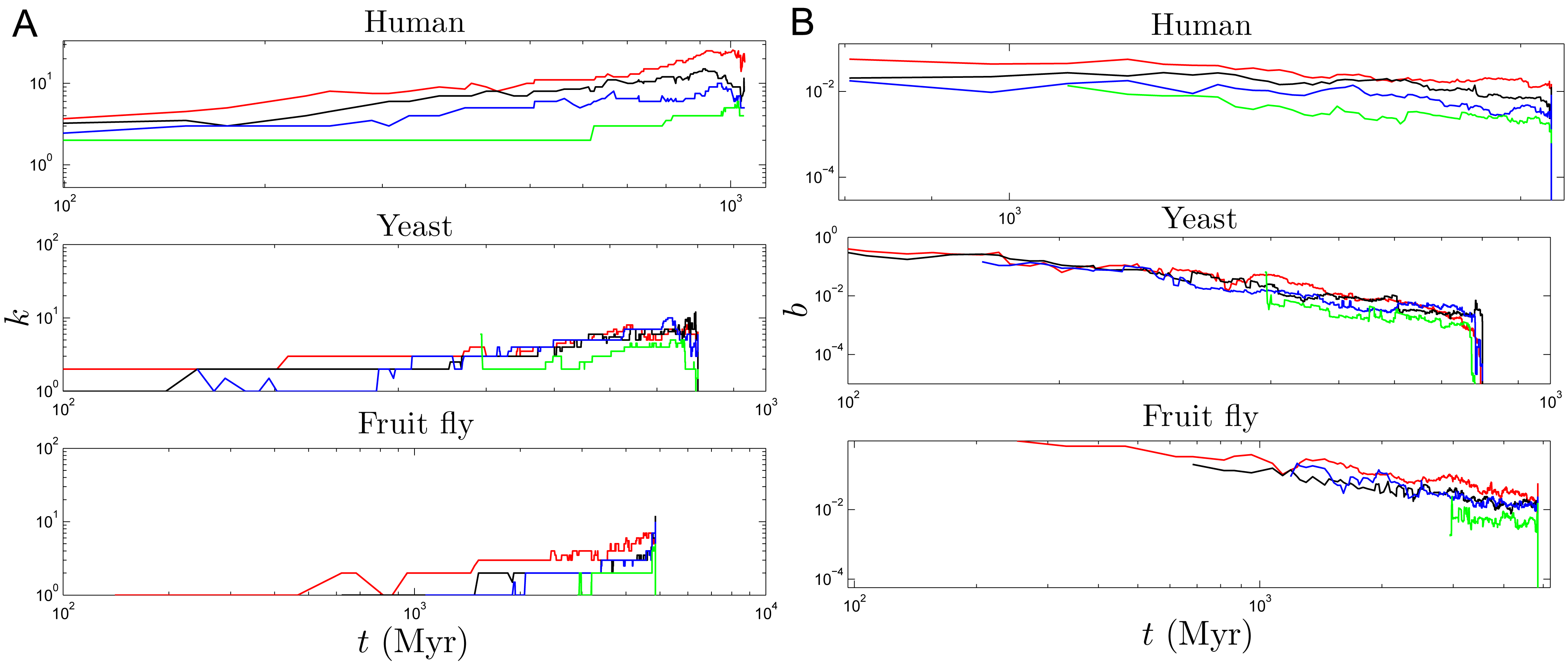

Supplement: Figure S4 — Individual protein centrality scores. Evolution of degree (A) and betweenness (B) for proteins introduced to the network at different times in humans (top), yeast (middle), and flies (bottom). The 1st protein (one of the two initial proteins) is shown in red, the 6th protein in black, the 11th protein in blue, and the 101st protein in green. Curves are median values from 50 simulations. (TIF) [file pone.0039052.s005.tif]

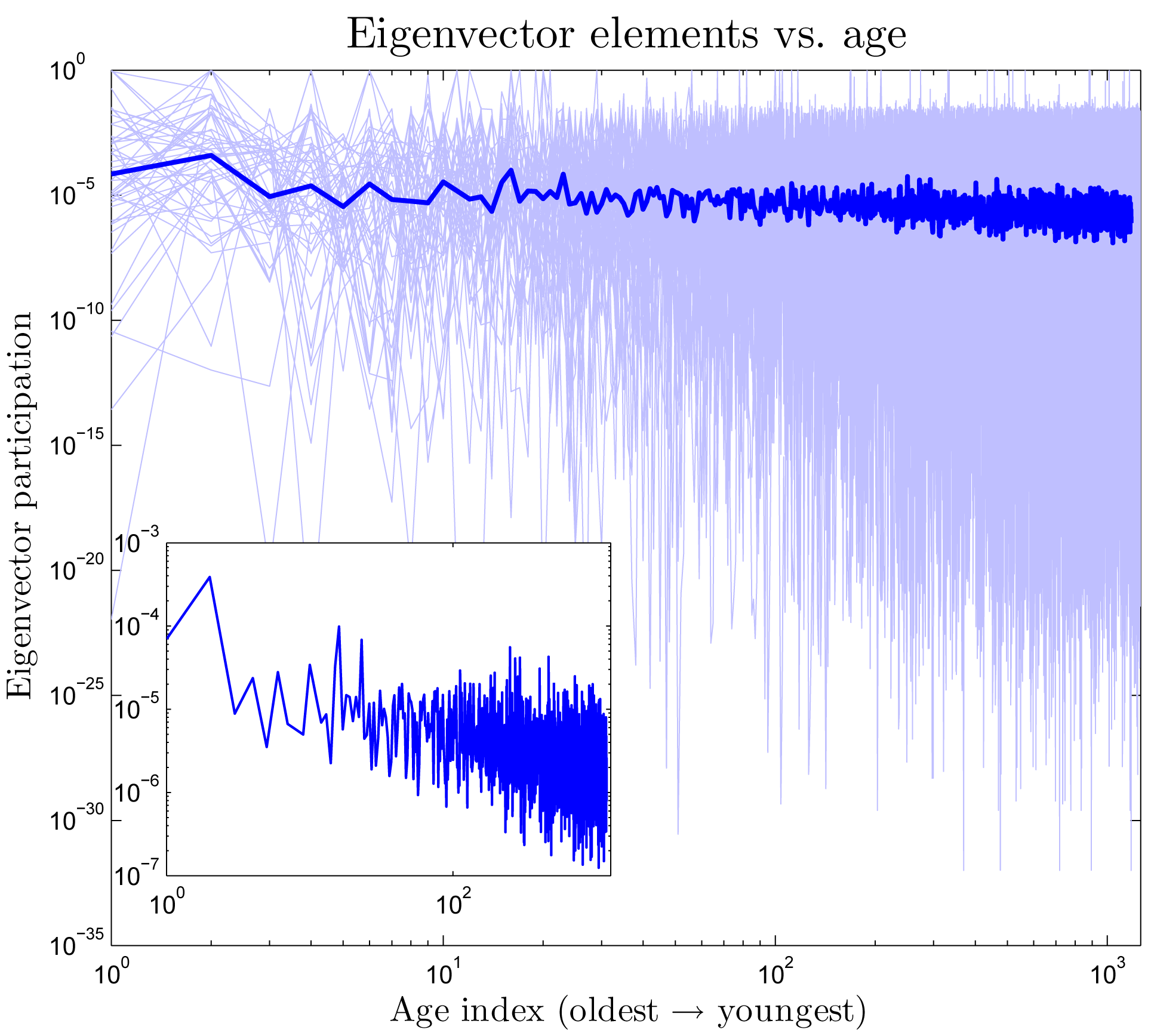

Supplement: Figure S5 — Laplacian eigenvector participation. Elements of the eigenvector of the Laplacian matrix (defined as , where is a diagonal matrix with the degree of node as element ) associated with the largest eigenvalue vs. protein age index (time of introduction) in the yeast simulation. Details of this method are discussed in [73]. Heavy lines are the median values from 50 simulations, and light lines are results of individual simulations. The inset plot shows the trend line with a rescaled -axis. (TIF) [file pone.0039052.s006.tif]

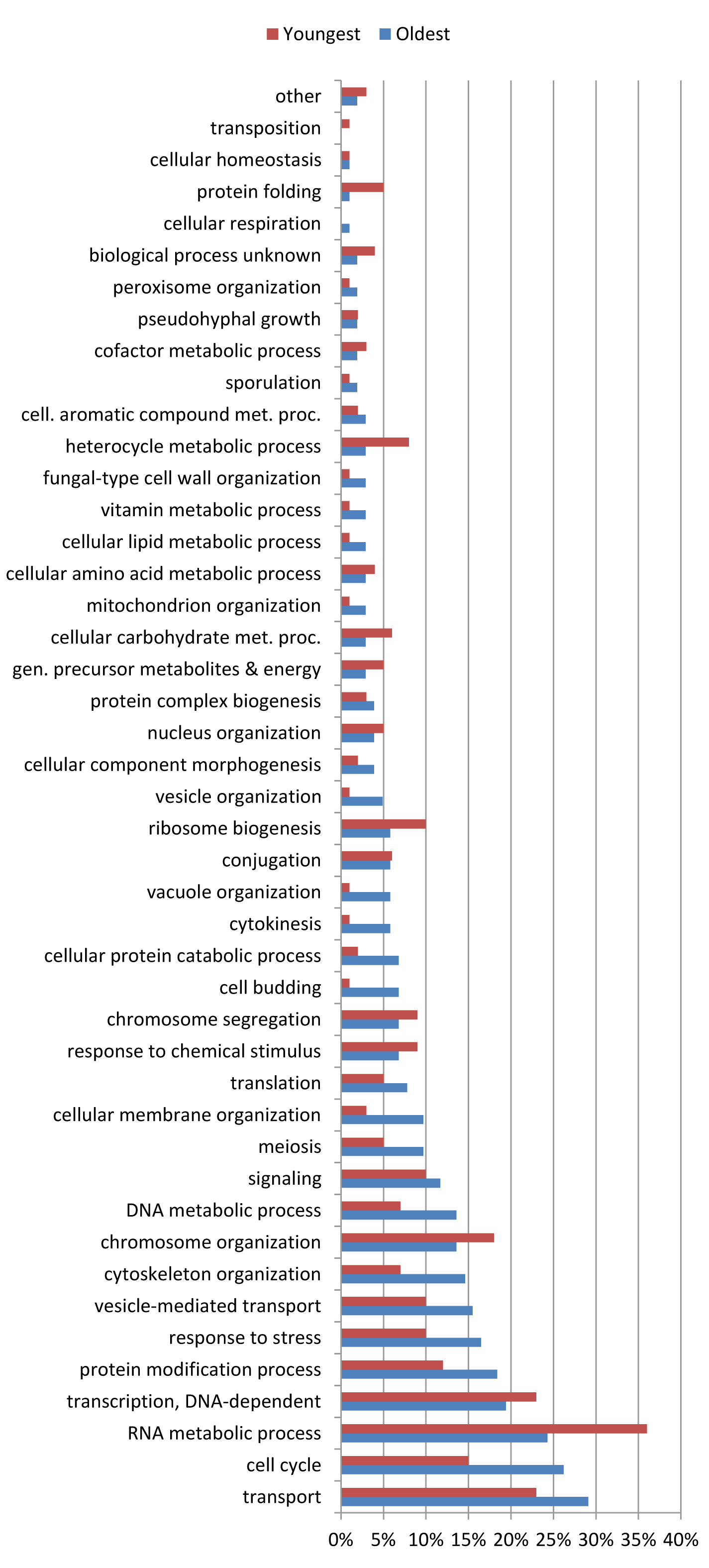

Supplement: Figure S6 — GO-slim profiles. Shown are profiles for the 100 oldest and 100 youngest proteins, as measured by -value, in the yeast PPI network. (TIF) [file pone.0039052.s007.tif]

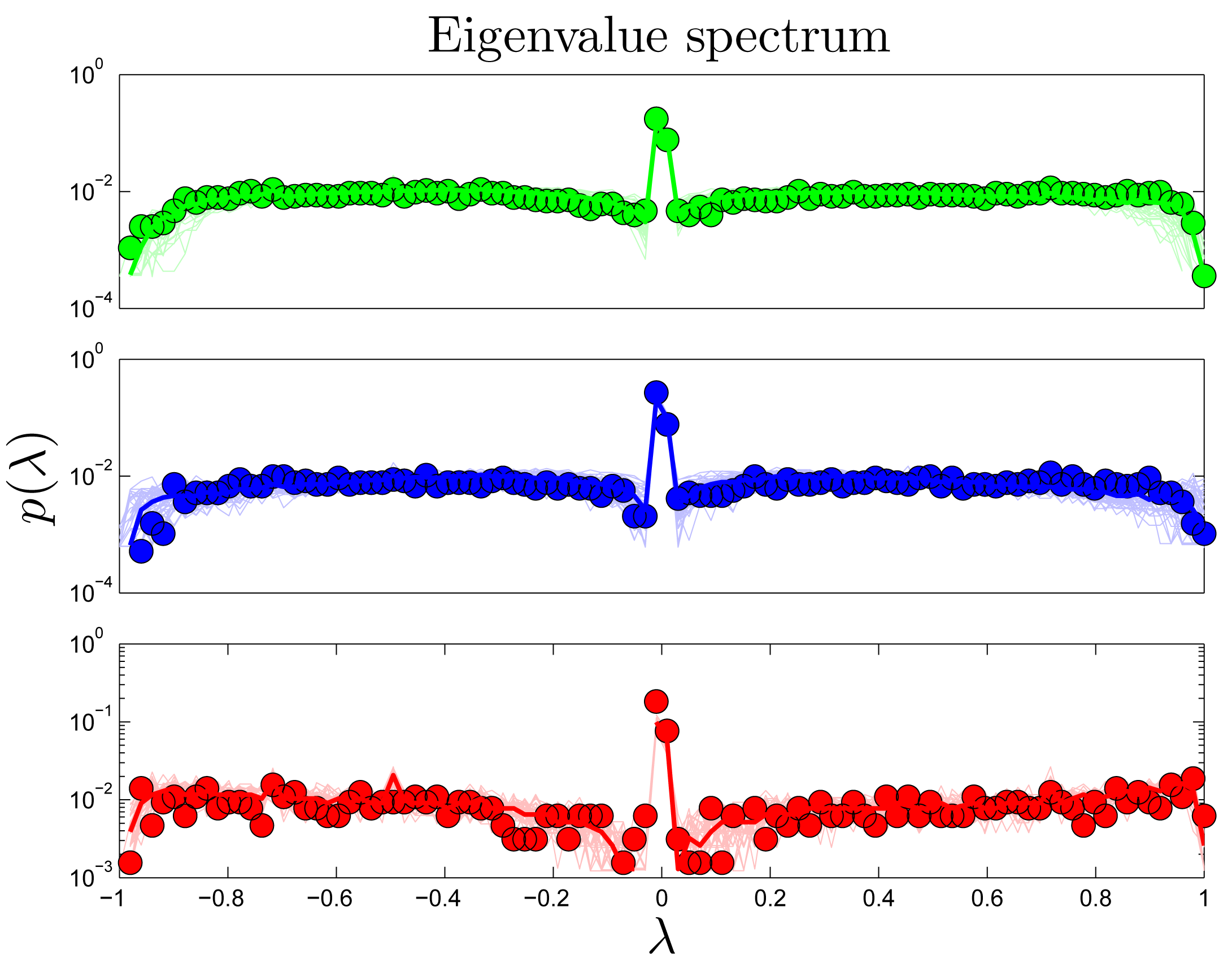

Supplement: Figure S7 — Walk matrix eigenvalues. Shown are eigenvalue () distributions in human (green), yeast (blue), and fly (red). Heavy lines are the median values from 50 simulations, and light lines are results of individual simulations. (TIF) [file pone.0039052.s008.tif]

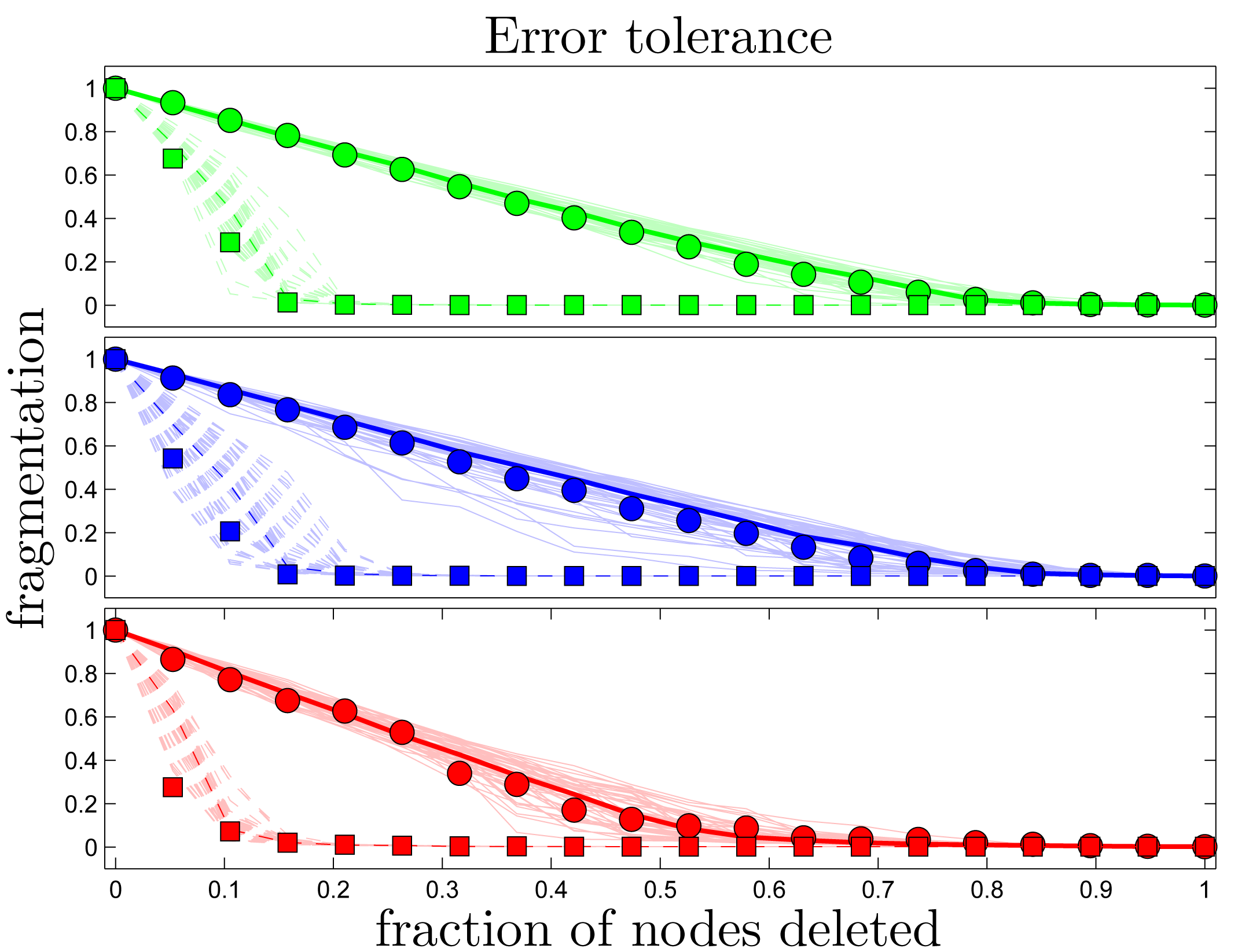

Supplement: Figure S8 — Error tolerance. Shown are error tolerance curves in human (green), yeast (blue), and fly (red). Circles indicate proteins deleted randomly, and squares indicate proteins deleted starting with the most well-connected protein and removing proteins in descending order. (TIF) [file pone.0039052.s009.tif]

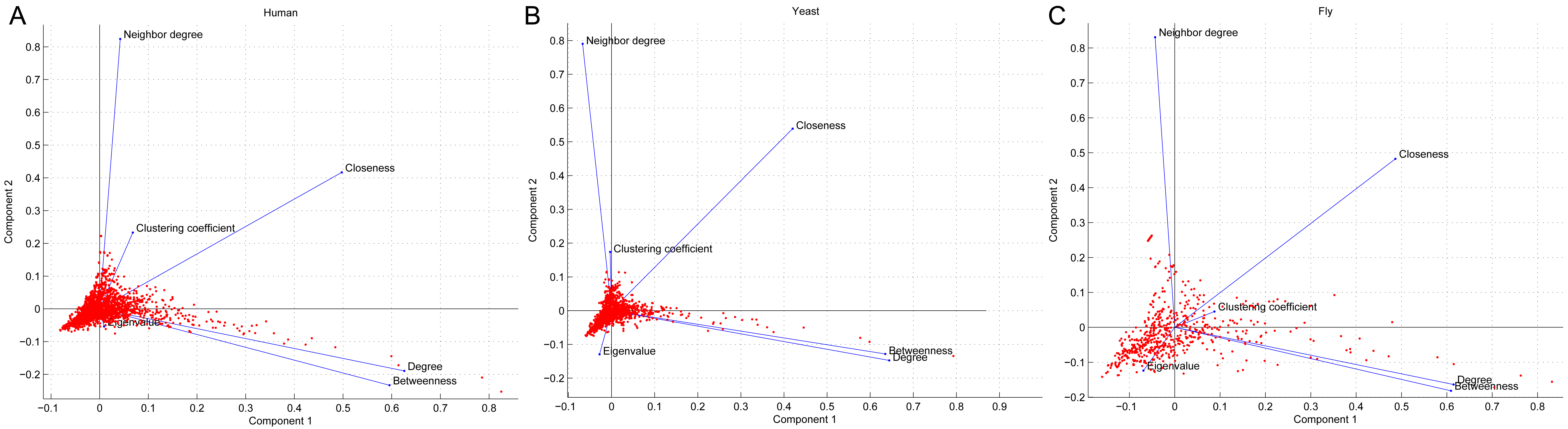

Supplement: Figure S9 — Principal component analysis. Shown are the factor loadings and scores on the first two principal components. Data scores are shown in red, and blue lines represent feature loadings. (TIF) [file pone.0039052.s010.tif]

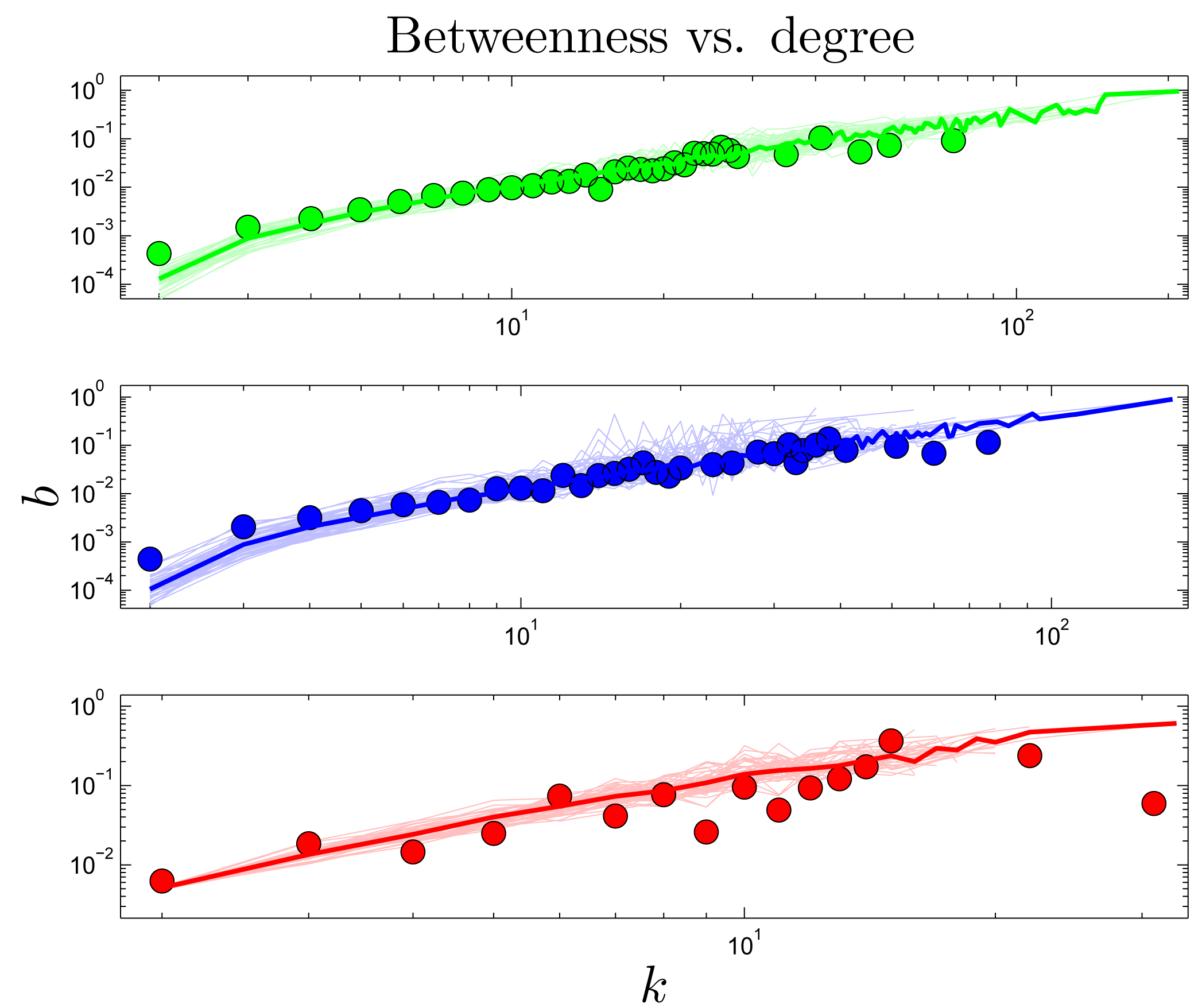

Supplement: Figure S10 — Betweenness vs. degree. Shown are median betweenness vs. degree values in human (green), yeast (blue), and fly (red). Heavy lines are the median values from 50 simulations, and light lines are results of individual simulations. (TIF) [file pone.0039052.s011.tif]

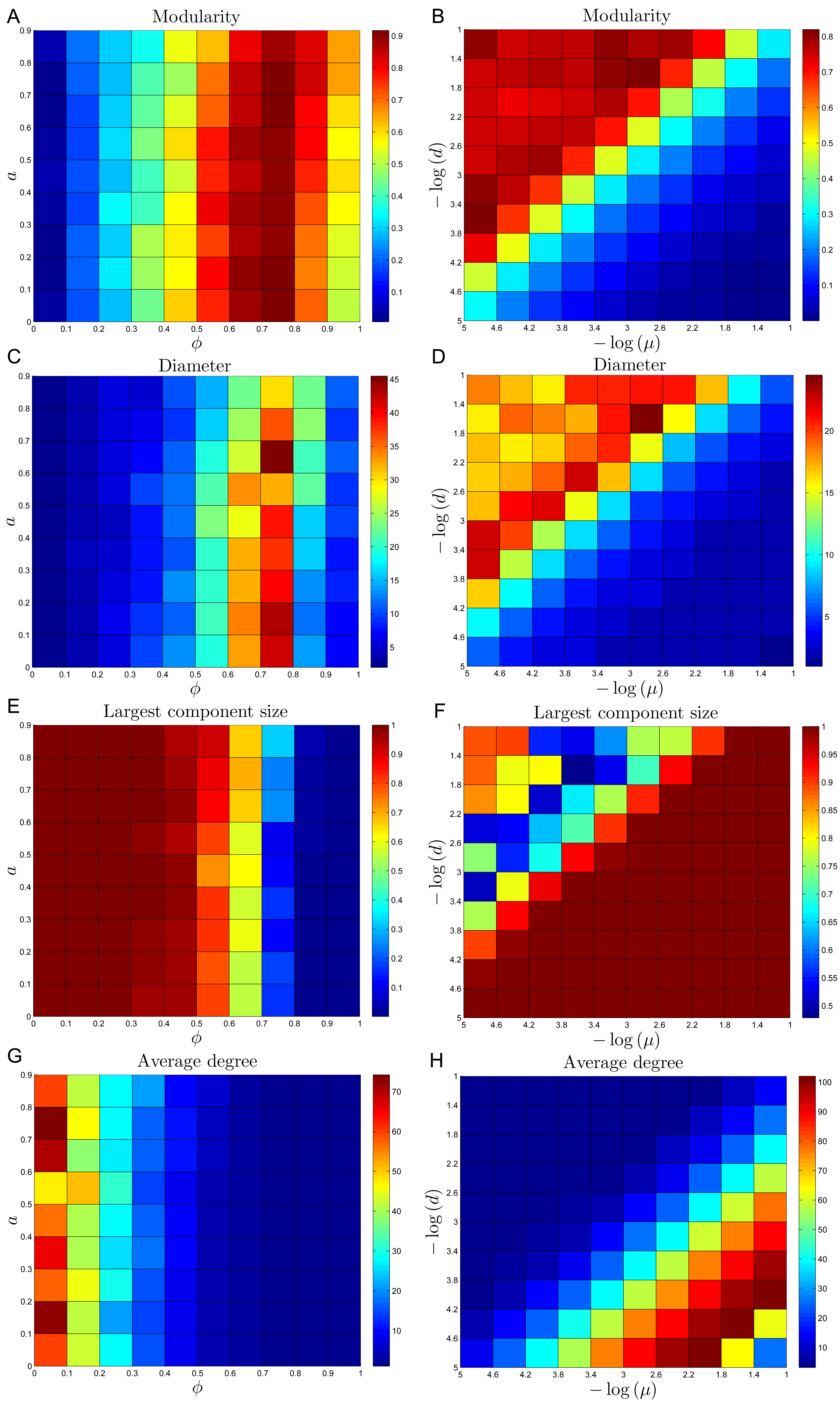

Supplement: Figure S11 — Sensitivity analysis. Heat maps represent median values for 10 simulations per parameter combination of the yeast network. Left: and are varied, and values are kept fixed. Right: and varied, and kept fixed. (TIF) [file pone.0039052.s012.tif]

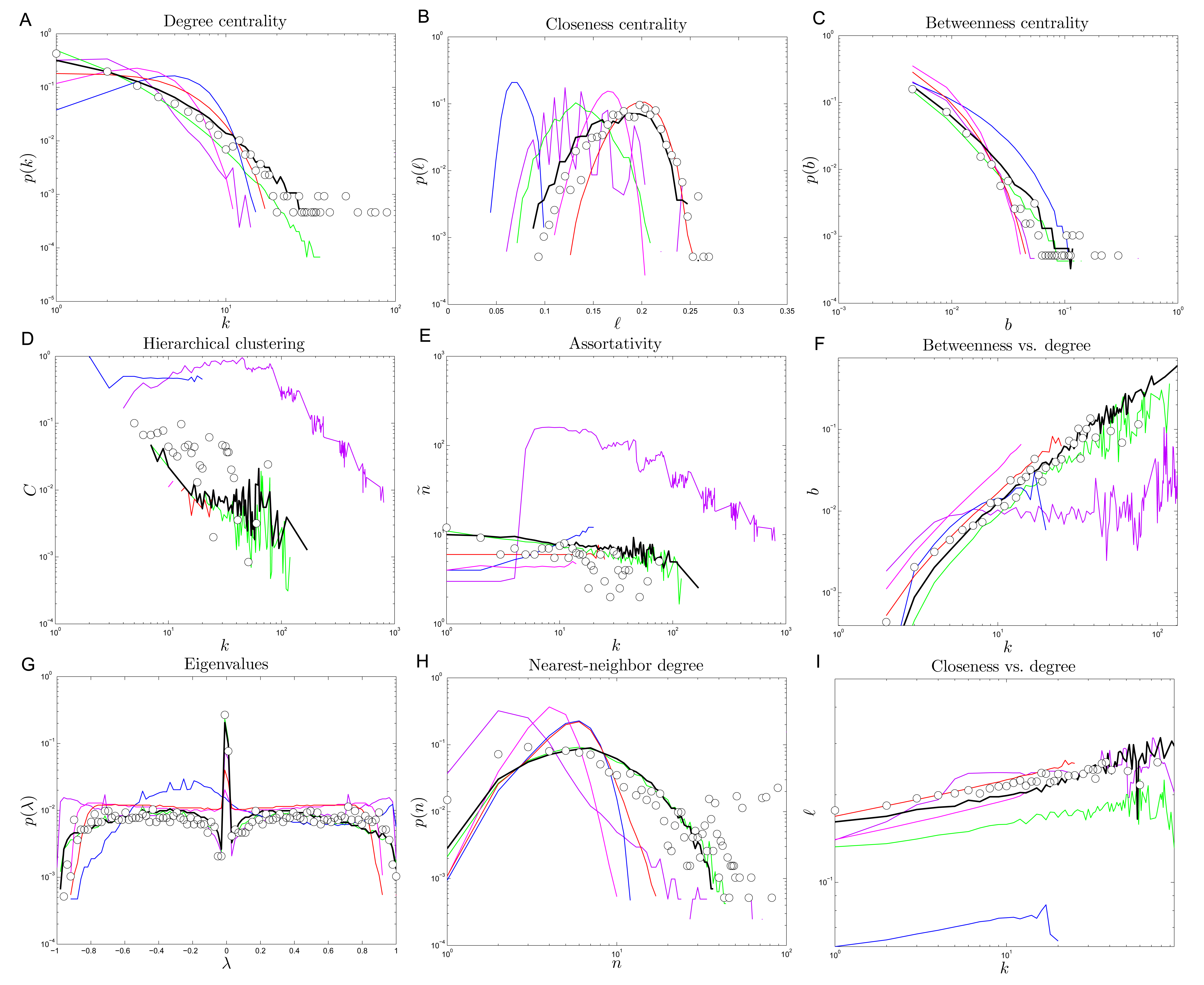

Supplement: Figure S12 — Model comparison. Comparison of five other models to the yeast PPI network: Vázquez [29] (green), Berg [85] (red), random geometric [89] (dark blue), MpK desolvation [52] (purple), and ER random graph [90] (brown). For reference, DUNE model results are shown as a black line. Dots represent high-confidence experimental yeast data, and solid lines are median values over 50 simulations. (TIF) [file pone.0039052.s013.tif]
