# Supplementary material for: Simulated Evolution of Protein-Protein Interaction Networks with Realistic Topology
Source: PLoS One. 2012 Jun 29;7(6):e39052. doi: 10.1371/journal.pone.0039052 (PMC3387198; doi:10.1371/journal.pone.0039052)
Supplement: Table S1 — Scaling exponents. Distributional exponents (, ) were estimated using the maximum likelihood method of [91]. Other exponents (, , ) were estimated using nonlinear regression. Due to the relatively small sizes of the data sets, there is considerable uncertainty in these estimates. (PDF) [file pone.0039052.s014.pdf]

Table S1. Scaling exponents.

	γ	β	ξ	α	δ
Yeast	2.8(2)	2.4(1)	1.8(2)	1.2(2)	0.32(6)
Fly	3.1(2)	2.2(4)	0.8(5)	0.8(7)	0.0(3)
Human	2.8(1)	2.3(1)	1.5(3)	1.3(2)	0.0(1)

Distributional exponents ($p(k) \sim k^{-\gamma}$, $p(b) \sim b^{-\beta}$) were estimated using the maximum likelihood method of [90]. Other exponents ($C \sim k^{-\xi}$, $b \sim k^{\alpha}$, $n \sim k^{-\delta}$) were estimated using nonlinear regression. Due to the relatively small sizes of the data sets, there is considerable uncertainty in these estimates.
