# Supplementary material for: Simulated Evolution of Protein-Protein Interaction Networks with Realistic Topology
Source: PLoS One. 2012 Jun 29;7(6):e39052. doi: 10.1371/journal.pone.0039052 (PMC3387198; doi:10.1371/journal.pone.0039052)
Supplement: Table S2 — SMAPE values. Symmetric mean absolute percentage error (SMAPE) of simulation versus experiment in yeast (Eq. ??). ‘E.T.’ is the error tolerance curve with random protein removal, and ‘E.T. ()’ is the error tolerance curve with highest-degree proteins removed first. ‘DUNE’ is the model described here, ‘Vázquez’ is the DU-only model of [29], ‘Berg’ is the link dynamics model [85], ‘RG’ is random geometric [89], ‘MpK’ is the physical desolvation model presented in [52], and ‘ER’ is an Erdös-Rényi random graph [90]. For each comparison, the lowest value is shown in bold. (PDF) [file pone.0039052.s015.pdf]

Table S2. SMAPE values

	$p(k)$	$p(\ell)$	$p(b)$	$C(k)$	$n(k)$	$b(k)$	$p(\lambda)$	E.T.	E.T. (k)	$p(n)$	$\ell(k)$	Total
DUNE	**0.47**	**0.37**	**0.36**	0.60	0.29	0.24	0.14	0.10	0.20	0.56	0.14	**3.48**
Vázquez	0.52	0.81	0.39	0.62	0.27	0.34	**0.12**	0.10	**0.15**	**0.50**	0.26	4.11
Berg	0.70	0.45	0.72	0.67	**0.15**	**0.19**	0.29	0.12	0.30	0.86	**0.06**	4.51
RG	0.81	0.98	0.61	0.83	0.22	0.23	0.33	0.31	0.45	0.89	0.49	6.15
MpK	0.82	0.79	0.68	0.89	0.88	0.58	0.18	0.18	0.18	0.82	0.16	6.18
ER	0.83	0.70	0.78	**0.53**	0.22	0.41	0.28	**0.07**	0.32	0.93	0.07	5.14

Symmetric mean absolute percentage error (SMAPE) of simulation versus experiment in yeast (Eq. 11). 'E.T.' is the error tolerance curve with random protein removal, and 'E.T. (k)' is the error tolerance curve with highest-degree proteins removed first. 'DUNE' is the model described here, 'Vázquez' is the DU-only model of [29], 'Berg' is the link dynamics model [84], 'RG' is random geometric [88], 'MpK' is the physical desolvation model presented in [52], and 'ER' is an Erdős-Rényi random graph [89]. For each comparison, the lowest value is shown in bold.
